# Comparative Study of Membrane Fouling with Aeration Shear Stress in Filtration of Different Substances

**DOI:** 10.3390/membranes13110867

**Published:** 2023-11-01

**Authors:** Weihao Yao, Bing Wang, Kaisong Zhang

**Affiliations:** 1 Key Laboratory of Urban Pollutant Conversion, Institute of Urban Environment, Chinese Academy of Sciences, Xiamen 361021, China; 2 University of Chinese Academy of Sciences, Beijing 100049, China; 3 College of Environmental Science and Engineering/Sino-Canada Joint R&D Centre for Water and Environmental Safety, Nankai University, Tianjin 300071, China; bwang@nankai.edu.cn; 4 College of Environmental Science and Engineering, Ocean University of China, Qingdao 266100, China; zhangkaisong@ouc.edu.cn; 5 Key Laboratory of Marine Environment and Ecology, Ministry of Education, Ocean University of China, Qingdao 266100, China

**Keywords:** FSMBR, CFD, aeration, shear stress, fouling rate, different substances

## Abstract

The formation process of membrane fouling is complex and diverse, which is an important problem that needs to be overcome in membrane applications. In this paper, three foulant systems consisting of humic acid, humic acid plus Ca^2+^ and humic acid plus Ca^2+^ plus yeast were selected to compare membrane fouling processes with different aeration intensities. The aim was to establish the quantitative relationship between membrane fouling rate and shear stress, respectively, in a large-scale flat sheet MBR (FSMBR). The shear stress values at different aeration intensities were obtained using computational fluid dynamics (CFD). The membrane fouling rate during the filtration of different substances was measured by performing experiments. The comparison results showed that the membrane fouling rate varied greatly during the filtration of different substances. With the help of particle size distribution, the effect of different shear forces on floc size was further explored. Using the dual control of fouling rate and floc size, the recommended aeration intensity was 6~8 L/(m^2^·min).

## 1. Introduction

Membrane fouling is an important factor restricting membrane application. Due to complex fouling mechanisms and processes, it has been a great challenge to overcome. Many methods have been used to mitigate membrane fouling, such as coagulation [1], adsorption [2], cleaning [3] and aeration [4]. Aeration has been proved to be a common means to control membrane fouling [5], but the deep mechanism of aeration to alleviate membrane fouling has not been thoroughly studied.

We have made some attempts to investigate the relationship between aeration and membrane fouling. Firstly, previous experiments investigated different aeration intensity in the filtration of yeast solution for the case of a single foulant, which revealed the quantitative relationship between shear stress and membrane fouling rate in physical particle fouling. It deepened the understanding of the mechanism of controlling membrane fouling. However, there are still some differences between the actual foulants and these simulated foulants. Considering that yeast is a particle with high hardness, the particle shape and size does not change much during aeration [6]. However, activated sludge is the actual filtration substance in a membrane bio-reactor (MBR); a large number of reports have shown that excessive shear stress will cause floc fragmentation [7]. Therefore, there is a gap between them. A scientific question is whether the quantitative relationship between shear stress and membrane fouling rate established in yeast systems was still applicable to other systems or complex systems.

The formation process of membrane fouling is usually complicated. Membrane foulants are usually complex mixtures. Some studies had reported that a variety of foulants could cause membrane fouling, and the membrane fouling caused by different types of foulants varied greatly [8]. In order to measure the contribution of different foulants to membrane fouling, it is, therefore, necessary to compare the differences among different foulants to obtain a comprehensive understanding of membrane fouling. Common membrane foulants are searched and summarized. Dissolved organic matter (DOM) is one of the main factors causing membrane fouling, and the main component is humic acid (HA) [9]. Ions and particles are widely found in sewage, and they will combine with humic acid (HA) to elevate the membrane fouling in ultrafiltration [10,11]. In addition, mixtures can cause more complex membrane fouling. Jermann et al. analyzed the mixed fouling layer formed on the surface of ultrafiltration membrane by natural organic matter (NOM) and kaolin [10]. Compared with single inorganic particles, mixtures will form more severe membrane fouling. Tahari et al. found that the relative concentration of humic acid would affect the density of cake [12]. In a word, there was interaction between the various foulants in a mixed system [13]. This kind of membrane fouling was defined as “mixed particle fouling” [14]. It was caused by organic or inorganic colloidal and dissolved substances. Based on the analysis of the filtering process, the cake skeleton formed by inorganic particles can be employed as a secondary membrane, which will function in pre-filtrating or adsorbing dissolved substances to change the cake property [15,16]. This leads to differences in membrane fouling. Compared with the single yeast solution, the formation and removal of cake are usually simultaneous and indistinguishable in the process of dynamic filtration [16]. The composition and size distribution of mixed particles becomes more complex. Because of the complexity of the filtration system, there is still a lack of quantitative research about mixed particle membrane fouling on the shear stress and fouling rate.

Therefore, the aim of this study is to compare the different effects of aeration hydrodynamics on membrane fouling with different filtration substances. Most of the current research on membrane fouling focuses on laboratory scale situations, which is far removed from industrial application. Large-scale industrial FSMBR systems were established for different foulants experiments. This enabled the results to be directly referenced and used by the industry. In addition, the particle size changes in different systems were paid attention to. The membrane fouling resistance and rate at different aeration intensities were combined with the change in particle size. The shear stress values were obtained using the verified CFD model. These were all investigated and evaluated in order to study the filtration and fouling processes from a detailed perspective. The relationship between fouling rate and shear stress in different systems was investigated. Establishing shear stress efficiency under mixed systems has an important guiding value for membrane fouling mitigating mechanisms. In addition, a deeper understanding of membrane fouling mechanisms can be developed as the basis for effective aeration strategies.

## 2. Methods

### 2.1. Experimental Study

#### 2.1.1. Large-Scale FSMBR System

**Figure 1:**
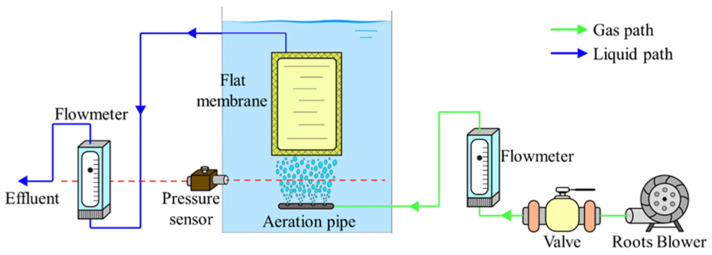
Schematic diagrams for fouling experiment in large-scale FSMBR.

Experiments were performed in an industrial-scale FSMBR system consisting of a rectangular tank with an operating volume of 16 m^3^ and with 20 flat sheets membranes (Oxiamembrane Co., Ltd., Xiamen, China). The schematic for the MBR is shown in Figure 1.

The PVDF flat sheet membrane was 1450 × 510 × 6 mm (H × W × D), with average pore size of 0.2 μm (contact angle of 80°) and effective filtration area of 1.25 m^2^ per membrane [17]. Channel gap between every two membranes was fixed at 8 mm as recommended by Oxiamembrane Co., Ltd. The free bubbling process was achieved using compressed air directed into the membrane tank with air flow rate of 4~12 L/(m^2^·min). Effluent flow rate was recorded every 1 s with paperless recorder (Sinomeasure, SIN-R200T, Hangzhou, China).

For all experiments, the water level was kept constant. The pump returned the effluent to the tank automatically. The static pressure difference between the pressure sensor and water surface was 30.0 kPa. The driving pressure calculated was 6.7 kPa. All experiments were conducted at room temperature.

The detailed dimensions for MBR system are described below. The aeration pipe (O.D. 20 mm) was placed in the bottom of tank to generate bubbles. On the top of aeration pipe, free bubbles were generated using four gas sparging nozzles with diameter of 5 mm and hole interval of 25 mm. The height between aeration pipe and membrane bottom was fixed at 400 mm.

In this experiment, HA, HA + Ca^2+^ and HA + Ca^2+^ + yeast were selected as the foulant systems for mixed particle fouling and the operation time was 3 days (Table 1). With reference to the practical concentrations of humic acid and ion in domestic sewage in MBR [18], the concentration of foulants was selected: humic acid (FA ≥ 90%, Macklin, Shanghai, China) 50 mg/L, calcium chloride dihydrate(CaCl_2_·2H_2_O, AR, Macklin, Shanghai, China) 5 mM and yeast (Angel, Yichang, China) 10 g/L.

The preparation methods for the three system solutions are as follows:

- Humic acid solution: mixing 500 g humic acid with 10,000 kg tap water in a water tank for more than 2 h using a gas with a maximum aeration intensity of 24 L/(m^2^·min).
- Humic acid + Ca^2+^ solution: mixing 500 g humic acid and 7350 g calcium chloride dihydrate with 10,000 kg tap water in a water tank for more than 2 h using a gas with a maximum aeration intensity of 24 L/(m^2^·min).
- Humic acid + Ca^2+^ + yeast solution: mixing 500 g humic acid, 7350 g calcium chloride dihydrate and 100 kg yeast with 10,000 kg tap water in a water tank for more than 2 h using a gas with a maximum aeration intensity of 24 L/(m^2^·min).

The particle size distribution of different systems was carried out with particle size meter (Malvern, Mastersizer 3000, Malvern, UK). The fouling rate of different aeration intensities in the different substances was emphatically explored.

#### 2.1.2. Data Collection and Analysis

According to Darcy:

(1) $$J=\frac{Q}{AT}$$

(2) $$J=\frac{p}{\mu \left(R_{m}+R_{f}\right)}$$

(3) $$R_{f}=\frac{p}{\mu J}-R_{m}$$

where *Q* is the osmotic volume at a certain time, m^3^

*A* is the membrane area, m^2^
*T* is the filtering time, s
*J* is the membrane flux, m^3^/ (m^2^·s)
*P* is the constant filtration pressure difference, kPa
*μ* is the viscosity of the transmission fluid, Pa·s
*R_m_* is the intrinsic resistance of membrane system, m^−1^
*R_f_* is the membrane fouling resistance, m^−1^

Cumulative water production volume is shown as

(4) $$V=\sum_{i=1}^{n}Q_{i}\Delta t$$

*V* is the cumulative effluent volume, m^3^
*Q_i_* is the instantaneous effluent flow, m^3^/h
Δ*t* is the recording interval, h.

### 2.2. Numerical Simulation Method

#### 2.2.1. Physical Model and Meshing

The hydrodynamics of the free bubbling process and its induced shear stress on each membrane surface were studied using computational fluid dynamics (CFD) simulations. A symmetric three-dimensional MBR geometry was set in GAMBIT 2.4.6 software.

The mesh contained 1,931,880 cells, 6,090,658 faces and 2,228,718 nodes. Average EquiSize Skew parameters of mesh were in the range of 0.2 to 0.3 (0–best and 1–worst), which indicated high-quality mesh and stable simulation running.

#### 2.2.2. Numerical Methods

A numerical model was conducted for calculation in ANSYS FLUENT 14.5. The VOF model was chosen here to simulate bubble motion behaviors since it is most suitable for interface tracking calculation [19]. Relative governing equations of mass momentum conservation were expressed as following:

(5) $$\frac{\partial \rho }{\partial t}+\nabla \left(\rho \vec{u}\right)=0$$

(6) $$\frac{\partial }{\partial t}\left(\rho \vec{u}\right)+\nabla \left(\rho \vec{u}\vec{u}\right)=-\nabla P+\rho \vec{g}+\rho \vec{F}+\nabla \vec{\tau }$$

where *P*, $\vec{g}$ and $\vec{F}$ are the pressure, gravitational acceleration and external force, respectively.

In general, density *ρ* and dynamic viscosity *μ* for an n-phase system were defined as

(7) $$\rho =\sum \alpha_{q}\rho_{q}$$

(8) $$\mu =\sum \alpha_{q}\mu_{q}$$

A number of turbulence models can be incorporated in the simulations; the realizable *k*–*ε* model was used to calculate for turbulence from bubbling process. The equations for *k* and *ε* were:

(9) $$\frac{\partial }{\partial t}\left(\rho k\right)+\frac{\partial }{\partial x_{j}}\left(\rho ku_{j}\right)=\frac{\partial }{\partial x_{j}}\left[\left(\mu +\frac{\mu_{t}}{\sigma_{k}}\right)\frac{\partial k}{\partial x_{j}}\right]+G_{k}+G_{b}-\rho \varepsilon -Y_{M}$$

(10) $$\begin{array}{l} \frac{\partial }{\partial t}\left(\rho \varepsilon \right)+\frac{\partial }{\partial x_{j}}\left(\rho \varepsilon u_{j}\right)=\frac{\partial }{\partial x_{j}}\left[\left(\mu +\frac{\mu_{t}}{\sigma_{\varepsilon }}\right)\frac{\partial \varepsilon }{\partial x_{j}}\right]+{\rho C}_{1}S\varepsilon -\rho C_{2}\frac{\varepsilon^{2}}{k+\sqrt{\upsilon \varepsilon }}\\ \;+C_{1\varepsilon }\frac{\varepsilon }{k}C_{3\varepsilon }G_{b}+S_{\epsilon } \end{array}$$

where

$$\mu_{t}=\rho C_{\mu }\frac{k^{2}}{\varepsilon }\;C_{1}=\max\;\left[0.43,\frac{\eta }{\eta +5}\right]\;\eta =S\frac{k}{\varepsilon }\;S=\sqrt{2S_{ij}S_{ij}}$$

where

$C_{\mu }$: function of the mean strain, rotation and turbulence fields;
$G_{k}$: turbulence kinetic energy due to the mean velocity gradients;
$G_{b}$: turbulence kinetic energy due to buoyancy;
$Y_{M}$: fluctuating dilatation in compressible turbulence to the overall dissipation rate;
$C_{2}$, $C_{1\varepsilon }$, $C_{3\varepsilon }$: constant;
$\sigma_{k\;}$: turbulent Prandtl numbers for *k*;
$\sigma_{\varepsilon }$: turbulent Prandtl numbers for *ε*;
$S_{k}$ and $S_{\varepsilon }$: user-defined source terms.

Surface tension of interface was simulated using continuum surface force (CSF) model [20], equations for which were expressed:

(11) $$p_{L}-p_{G}=\sigma k$$

(12) $${\underset{F}{\to }}_{vol}=\sigma \frac{2\rho \kappa \nabla_{\alpha_{G}}}{\left(\rho_{L}+\rho_{G}\right)}$$

(13) $$\kappa =\nabla \hat{n}$$

(14) $$\hat{n}=\frac{n}{\left|n\right|},\;n=\nabla \alpha_{q}$$

The pressure-based solver was chosen, and a second-order upwind scheme was used for momentum and *k*–*ε* equations discretization. PRESTO! (pressure staggering option) the scheme was chosen for the pressure term discretization. Different aeration intensities from 4 to 12 L/(m^2^·min) were applied in various calculation runs. The boundaries for the tank walls were all stationary and there was no fluid-slip condition at the membrane surface.

## 3. Results and Discussion

### 3.1. Size Distribution

**Figure 2:**
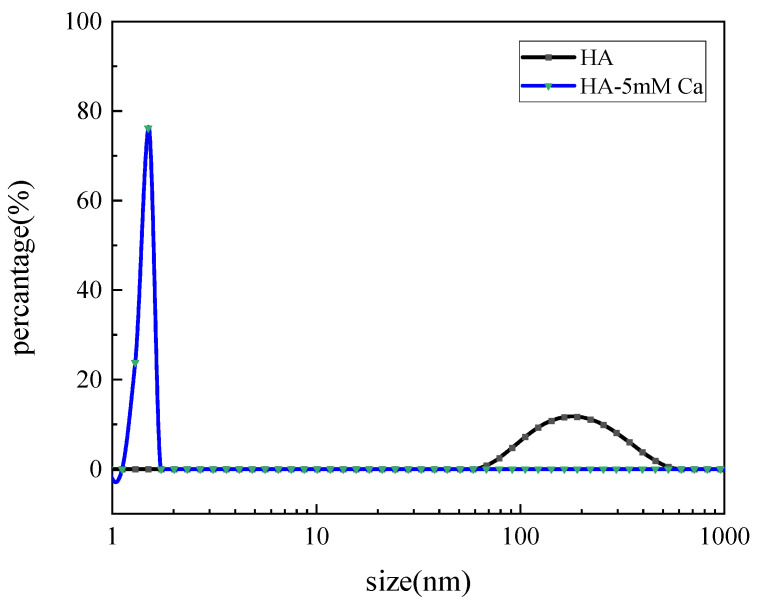
Comparison of particle size distribution after mixing humic acid and calcium separately.

Since microfiltration mainly relies on the pore size sieving effect, it is necessary to characterize the particle size distribution of the system. Some papers reported that specific physical parameters (size distribution and floc aggregation) were affected by the working conditions [21]. Therefore, the particle size difference between them can be used as a comparative object. In Figure 2, the particle size of humic acid was about 200 nm. The humic acid used is a chemical agent (FA ≥ 90%), slightly different from the actual humic acid with high mixture [22]. In the HA-5 mM Ca^2+^ system, the particle size decreased to less than 2 nm, the width of the peak decreased and the height increased. It can be seen that the particle size of HA changes greatly after the addition of Ca^2+^, which indicated that the particle size became smaller and the particle size range was more concentrated. Ca^2+^ reduced the size of humic acid particles, which resulted in the size distribution being more uniform. It has been reported that Ca^2+^ could compress the size of negatively charged HA molecules through the electrostatic shielding effect, thereby leading to the decrease in HA floc size [23].

**Figure 3:**
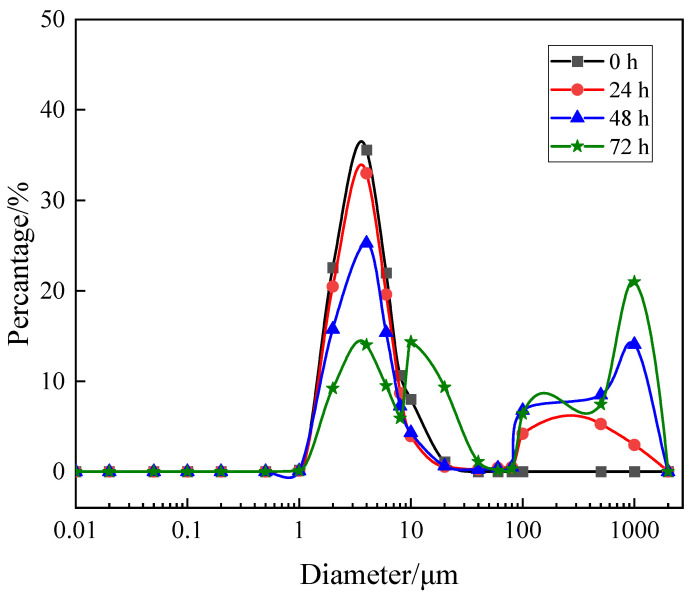
Particle size changes of mixed systems over different running times.

As can be seen in Figure 3, the particle size distribution of the mixed system has changed during operation. For the humic acid + Ca^2+^ + yeast system, there was only a specific size peak of yeast of about 4 μm at the initial stage, and then the peak size of 100~1000 μm was formed gradually (Figure 3). With the increasing running time, the proportion of flocs with large particle size gradually concentrated in the range of 100~1000 μm. At the increased running time (72 h), there were some components slightly larger than 10 μm. The whole system belonged to the mixed system with multi-particle size distribution. This can work in two ways: (1) HA can coagulate with particles [24] or (2) small particles are easily adhered to cake, while large particles are more easily affected by shear stress [25]. The change in particle size distribution needs to be noted in the next section on membrane fouling rate.

### 3.2. Flux Decline and Fouling Resistance

#### 3.2.1. Humic Acid

**Figure 4:**
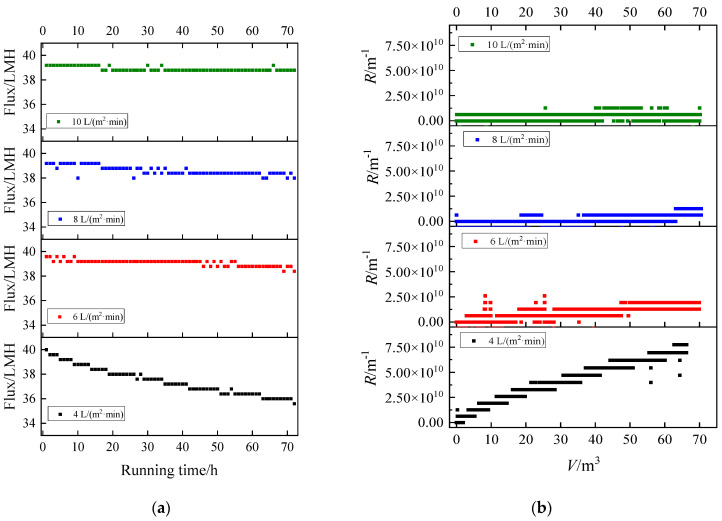
Fouling of humic acid under different aeration intensities: (**a**) flux vs. running time and (**b**) *R* vs. *V*.

For HA, the fouling rate was relatively slow (Figure 4). This indicated that single HA had a weak tendency to foul MF membranes. Even for the smallest intensity (4 L/(m^2^·min)), the flux decline was not more than 10% within 72 h. When the intensity gradually increased to 6 L/(m^2^·min), the decline rate of flux slowed down significantly, only decreasing by about 5%. However, for 8 and 10 L/(m^2^·min), the flux hardly declined and oscillated around the initial flux. Although the fouling rate was not fast, the mitigating effect on membrane fouling could still be distinguished. It was related to the fact that the particle size of HA was close to membrane pore size (200 nm). HA could show colloid properties, which could not easily lead to serious membrane fouling.

Except for the 4 L/(m^2^·min), the membrane resistance curves were similar and the final *R* was within 2 × 10^10^ m^−1^. But the resistance curve had obvious climbing phenomenon at 4 L/(m^2^·min), and the final *R* could reach 7.7 × 10^10^ m^−1^, which was 3.85 times that of others. At 4~6 L/(m^2^·min) aeration intensity, the membrane fouling rate changed obviously. However, the fouling rate at 6~10 L/(m^2^·min) was similar. This reflected that when the membrane fouling rate was slow, the difference at different aeration intensities was not significant.

#### 3.2.2. Humic Acid + Ca^2+^

**Figure 5:**
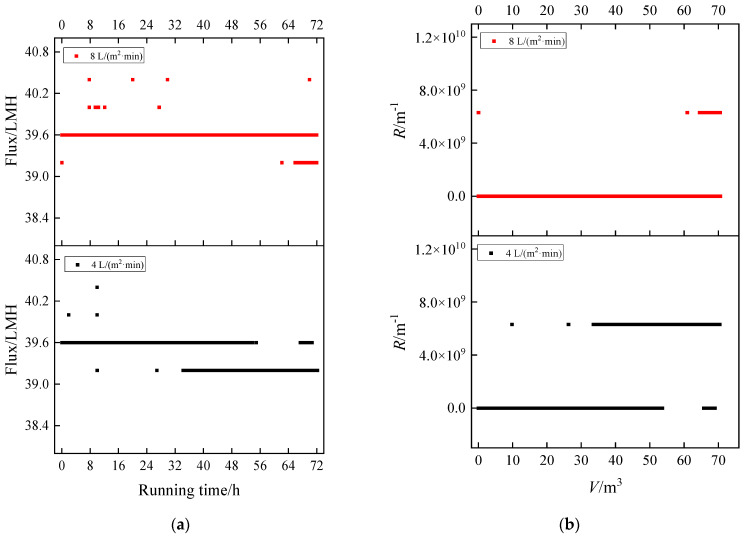
Fouling of humic acid and calcium under different aeration intensities: (**a**) flux vs. running time and (**b**) *R* vs. *V*.

For the mixed system of HA + Ca^2+^, no obvious flux decline occurred at 4 and 8 L/(m^2^·min) (Figure 5a). According to the particle size (<2 nm), the flocs can easily pass through the membrane pore (200 nm) without deposition on the membrane surface and pore. From the time that flux (from 39.6 to 39.2 LMH) decreased initially, the time was 32 h at 4 L/(m^2^·min), and 64 h at 8 L/(m^2^·min). The fouling rate of 8 L/(m^2^·min) was slightly slower than that of 4 L/(m^2^·min), but for both membranes the fouling was very weak. The same trend can also be seen in the *R* vs *V* (Figure 5b). The resistance rose slowly and the final resistance did not reach 8 × 10^9^ m^−1^. Compared to the MF pore (0.2 μm), the membrane fouling rate of the HA + Ca^2+^ system was very weak, so no further exploration of other aeration intensities was carried out. Furthermore, for UF, HA + Ca^2+^ may cause serious membrane fouling, while for MF with a larger pore size, the fouling rate was extremely weak.

#### 3.2.3. Humic Acid + Ca^2+^ + Yeast

**Figure 6:**
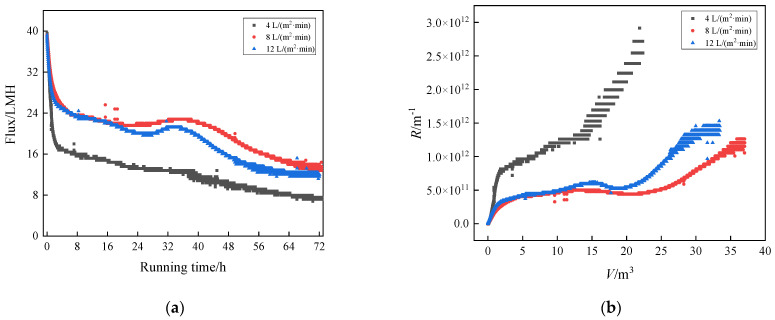
Comparison of fouling in complex systems with different aeration rates: (**a**) flux vs. running time and (**b**) *R* vs. *V*.

In Figure 6, it can be seen that the membrane fouling process was relatively complex and interesting for the HA + Ca^2+^ + yeast system. The flux decline rates of the three curves were very fast in the early stage, but slowed down gradually (Figure 6). The flux of 4 L/(m^2^·min) declined most rapidly, which was easily understood. While the flux changes of 8 and 12 L/(m^2^·min) were interesting. In the early stage, the flux decline of the large intensity (12 L/(m^2^·min)) was slightly faster than that of the medium intensity (8 L/(m^2^·min)). In the middle stage, the flux decline of 12 L/(m^2^·min) was obviously faster than that of 8 L/(m^2^·min), and the two intensities showed a certain plateau (fluctuation), which showed a balanced contest between shear stripping and filtration fouling [26]. The terminal fluxes were similar; 8 L/(m^2^·min) was still slightly higher than 12 L/(m^2^·min). The abnormal phenomenon was also very similar to the relevant engineering experience in actual activated sludge systems. Excessive aeration intensity can cause the fragmentation of sludge flocs.

**Figure 7:**
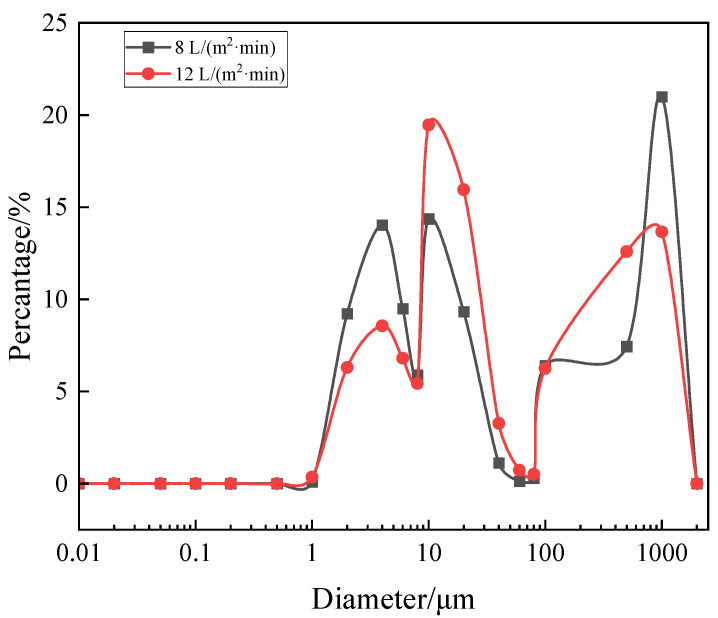
Comparison of particle size distribution of systems at 8 and 12 L/m^2^·min.

In order to explain this interesting result, we took samples and tested the particle size distribution of the solution at the end of filtration. As shown in Table 2, D [4, 3] represents the volume quadric moment mean diameter (average particle size). It can be seen from Figure 7 and Table 2 that when aeration intensity increased from 8 to 12 L/(m^2^·min), D [4, 3] value decreased from 414 to 308 μm. It was related to the reduction in large-size components (D90). This may be due to the fact that increased shear stress will break up the floc. There are reports that the cake void formed by large particles was bigger, and the filter cake void formed by small particles was smaller. This reflected the fact that the density of the filter cake layer was one of the things that affects membrane fouling. In addition, some studies had shown that the shear stress had a better effect on the peeling of large particles, while small particles were more likely to adhere to the cake. It indicated the difference in the effect of shear stress on different size particles. From the above two points, it can be inferred that the reduction in particle size plays a dominant role in the competition with increasing shear stress at 12 L/(m^2^·min).

### 3.3. Bubble and Shear Stress Distribution

**Figure 8:**
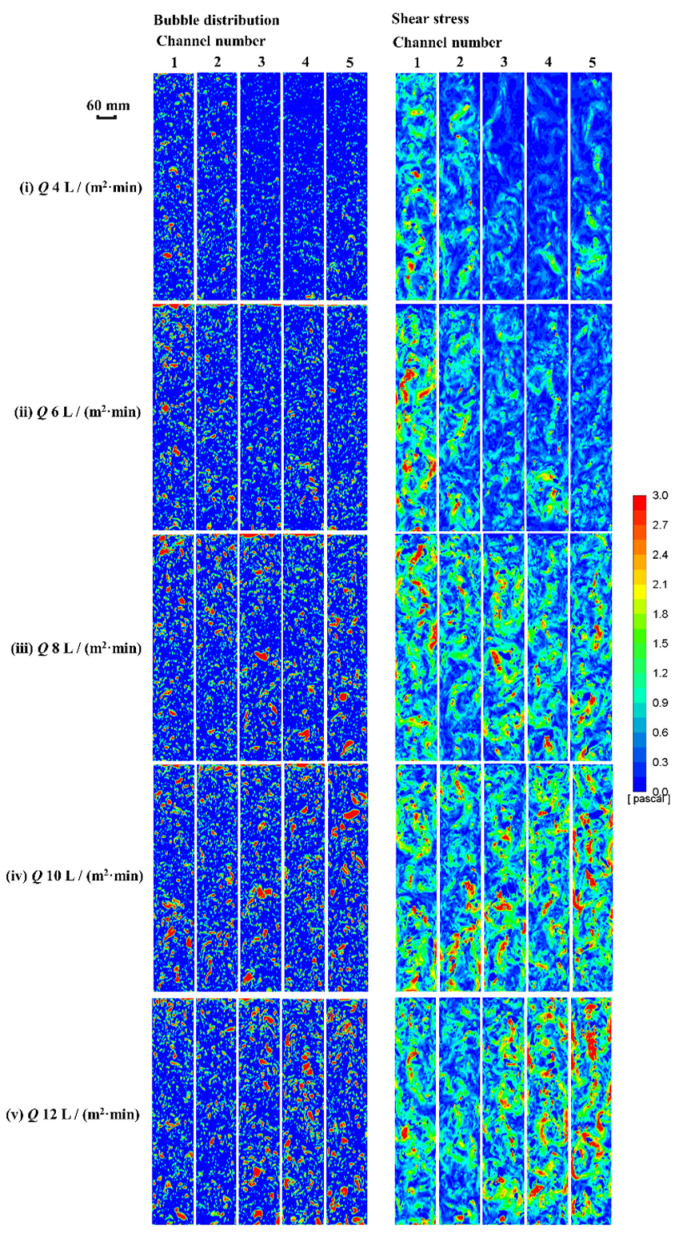
The distribution of bubble and shear stress.

The distribution of bubbles and shear stress between the two flats can be clearly seen from the CFD simulation results. Within a certain range, the shear stress was positively correlated with the volume of the bubble [27]. The bubbles and shear stress contours were shown at various aeration intensities. As shown in Figure 8, with the increasing intensity, the proportion of large bubbles increased and transferred to the outer channels gradually. When the intensity was 10 L/m^2^·min, the distribution of bubbles and shear stress was the most uniform. When the intensity was 12 L/m^2^·min, there were fewer bubbles in the middle channels and the large bubbles were mainly distributed in the outer three channels. This may be due to the poor orientation of large bubbles leading to the uneven distribution in the channels. The results of shear stress at various aeration intensities were summarized to facilitate the evaluation of the relationship between shear stress and aeration intensity (Table 3). Average and maximum shear stress were usually adopted as two characteristic factors. It can be seen from Table 3 that both of them increased with the rise of aeration intensity, and the maximum value was reached at 10 L/m^2^·min. When the aeration intensity increased to more than 10 L/m^2^·min, the bubble size and shear stress did not increase significantly or even decreased.

### 3.4. Fouling Rate and Shear Stress

**Figure 9:**
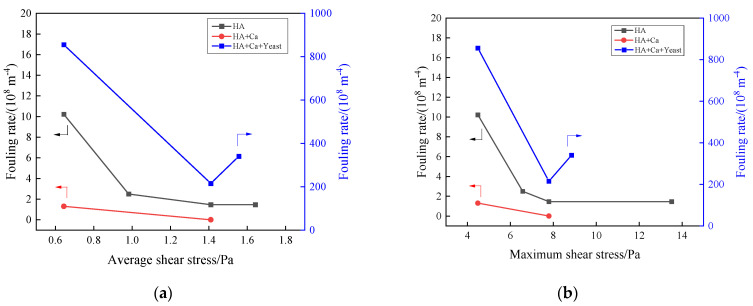
The relationship between shear stress and membrane fouling rate: (**a**) average shear stress and (**b**) maximum shear stress.

The experiment considers the shear stress of different aeration intensities as a factor in controlling membrane fouling. By linking shear stress with membrane fouling rate, the quantitative correspondence between the two can be more vividly demonstrated. The relationship between average and maximum shear stress and membrane fouling rate was similar (Figure 9). In the HA + Ca^2+^ system, no matter whether the intensity was set at 4 or 8 L/(m^2^·min), the fouling rate was also low (<2 × 10^8^ m^−1^). Although it can reflect the aeration mitigation effect of membrane fouling, the distinction was not obvious. For HA, in the range of 4~6 L/(m^2^·min), the membrane fouling rate decreased significantly and the trend slowed down gradually. For HA + Ca^2+^ + yeast, with the increase in aeration intensity, the membrane fouling rate increased first significantly and then slightly.

The particle size distribution changed during the filtration process within this experiment, and the change in particle size was included in the analysis of membrane fouling rate. When the aeration rate was larger, the increase in membrane fouling rate was related to the decrease in floc size (Table 2). It can be seen from the comparison of membrane fouling rates that HA, HA + Ca^2+^ and HA + Ca^2+^ + yeast are very different, the difference of fouling rate being more than 10 times. For HA + Ca^2+^, the floc was far smaller than the pore size (Figure 2), and most of them could directly passed through the membrane pore, which caused weak membrane fouling. While for HA + Ca^2+^ + yeast, the fouling was mainly caused by cake, so the fouling rate was fast. When the shear stress was too large, the large flocs break into small flocs. It can be seen that the optimal aeration intensity of the three systems was 6~8 L/(m^2^·min), which was consistent with the aeration intensity of 7 L/(m^2^·min) used in early research.

Large-scale FSMBR with 20 sheets membranes were constructed in this work, effectively eliminating the difference between lab-scale and industrial-scale. Thus, both the CFD simulation and the experiments were based on industrial-scale MBR, the results of which could be directly applied in industrial applications.

This experiment explored the membrane fouling under non-steady state (particle size variation) conditions, providing theoretical reference for practical engineering, especially during start-up and debugging periods.

## 4. Conclusions

From the continuous filtration experiment on 20 sheets industrial-scale flat membranes, the fouling rates of the three foulant systems were obviously different, which was related to the particle size distribution. According to the bubble simulation results of the CFD, the uniformity of aeration was crucial for controlling membrane fouling in industrial-scale membrane modules. In the HA + Ca^2+^ + yeast system, the fouling rate of medium aeration intensity (8 L/(m^2^·min)) was slightly slower than that of high aeration intensity (12 L/(m^2^·min)), which corresponds to the change in particle size distribution. It suggested that the mixed particle fouling was more complex than the single particle fouling. Considering the membrane fouling rate and floc size, the recommended aeration intensity was 6~8 L/(m^2^·min). The experiment provided an exploration of the impact of particle size changes on membrane fouling in mixed systems. It is hoped the method will be applied and validated in other systems.

## Figures and Tables

**Table 1:** Experimental parameter design and shear stress simulation values.

No.	Component	Aeration Intensity/(L/m^2^·min)
1	HA	4
2		6
3		8
4		10
5	HA + Ca^2+^	4
6		8
7	HA + Ca^2+^ + Yeast	4
8		8
9		12

**Table 2:** Special particle size at 8 and 12 L/m^2^·min.

Aeration Intensity/(L/m^2^·min)	D [4, 3]/μm	D10/μm	D50/μm	D90/μm
8	414	4.09	17.1	1500
12	308	4.75	22.3	1150

**Table 3:** Summary of shear stress at various aeration rates.

Aeration Intensity/(L/m^2^·min)	4	6	8	10	12
Average shear stress/Pa	0.644	0.982	1.410	1.642	1.556
Maximum shear stress/Pa	4.486	6.573	7.794	13.512	8.849

## Data Availability

Data are contained within the article.
